# Development and Validation of the Chinese Version of the Highly Sensitive Child Scale: Understanding environmental sensitivity and depressive symptoms in adolescents

**DOI:** 10.3389/fpsyt.2022.999150

**Published:** 2022-12-05

**Authors:** Qian Dong, Lina Zhou, Wei Wang, Xin Wei, Michael Pluess, Xiancang Ma

**Affiliations:** ^1^Department of Psychiatry, The First Affiliated Hospital of Xi’an Jiaotong University, Xi’an, Shaanxi, China; ^2^Xi’an No.3 Middle School, Xi’an, Shaanxi, China; ^3^Department of Biological and Experimental Psychology, School of Biological and Chemical Sciences, Queen Mary University of London, London, United Kingdom; ^4^Centre for Economic Performance, London School of Economics, London, United Kingdom

**Keywords:** environmental sensitivity, maternal behaviors, depressive symptoms, scale development, moderation, Chinese adolescents

## Abstract

**Background:**

Environmental sensitivity (ES) is considered a significant personality factor in the development and maintenance of depressive symptoms in adolescents. However, a clear instrument that can capture ES in Chinese adolescents is lacking. The current study aimed to investigate the psychometric properties of a Chinese version of the Highly Sensitive Child (HSC) Scale for assessing adolescent ES, and explore the potential moderation effect of ES on relationships between maternal behaviors and adolescent depressive symptoms.

**Methods:**

In total, 2,166 students from four middle and high schools and 105 depressed adolescents completed measurements of environmental sensitivity, maternal behaviors, depressive emotions, sleep duration, and academic performance.

**Results:**

First, exploratory factor and confirmatory factor analyses indicated that the HSC scale had a good model fit with the bifactor construct, total scale reliability was adequate-good, and measurement invariances across genders and different samples were supported. Furthermore, the results confirmed that the relationship between maternal behaviors and adolescent depressive symptoms had small effects. Compared to low environmentally sensitive adolescents, high environmentally sensitive adolescents exhibited less depressive emotions and better academic performance in the context of high-quality maternal behaviors. Low-quality maternal behaviors significantly predicted increased depressive emotions and worse academic performance in adolescents when environmental sensitivity was high. Moreover, on the contrary, maternal behaviors did not influence depressive emotions and academic performance in adolescents who were less sensitive to their environment. The relationship between maternal behaviors and adolescent depressive symptoms is influenced by different levels of environmental sensitivity.

**Conclusion:**

Our findings support the HSC scale as a comprehensive and psychometrically robust tool to measure ES in Chinese adolescents. In addition, the present study clarifies the moderating role of environmental sensitivity underlying the relationship between maternal behaviors and adolescent depressive symptoms. It is important to consider the role of ES in prevention and intervention strategies targeting adolescent depressive symptoms.

## Introduction

The incidence of depression begins to rapidly increase during the phases of adolescent development (1–3). According to a report on national mental health development in China (2019–2020), the diagnosis rate of depression among adolescents was 24.7%, with 17.2% of cases considered mild and 7.4% considered major (4); thus, a quarter of Chinese adolescents may have symptoms of depression. While literacy, prevention, and interventions for depression progress, rates of active professional help seeking in China remain low, especially for adolescents (5). Depression in adolescents is associated with a risk of suicide, school refusal behavior, and long-term poor psychosocial outcomes (6–8). However, compared to clinically diagnosed depression, depressive symptoms in adolescents are easier to recognize because it is less time-consuming and parents, teachers, and peers do not need to be highly trained to identify it. Most importantly, depressive symptoms are warning signs of adolescent depression. Thus, a push for a greater focus on which youths are most at risk or could benefit from surrounding environment changes, and investigation into the precursors and predictors of adolescent depression symptoms, are of great significance.

Prior studies have shown that adolescents respond differently to the same environmental impacts, either stressful or supportive, indicating that some adolescents are more affected by contextual factors than others (9–11). This capacity for environmental sensitivity (ES) in adolescents is defined as the ability to register and handle outside stimuli and is one of the most fundamental individual characteristics and personality traits (12–14). Many environmental factors, including social media, schools, and families, are key targets for early prevention in order to reduce depressive symptoms in adolescents, but preventive effects tend to differ along with varying levels of an adolescent’s environmental sensitivity (15–17). Previous research on the long-term effects of high levels of environmental sensitivity on adolescent development has been informed by a deficit perspective (18, 19); these results were more in line with environmental sensitivity acting as a vulnerability personality factor impacting depressive symptoms. Higher ES is associated with an increased risk of developing depression and anxiety. Recently, this area of research has found that high-sensitivity adolescents seem not only to be more affected by adverse exposure leading to problems in daily function and depressive symptoms, but also benefited more from positive environments increasing adolescent well-being and life satisfaction, whereas the low-sensitivity group were resilient to poor conditions and did not do exceptionally well in ideal ones (20–22). However, evaluating whether ES moderated the relationship between environmental factors and depressive symptoms in adolescents has yielded mixed results (23). It is vital to investigate individual differences in adolescent environmental sensitivity in order to determine which youths are at most risk or benefit across this core developmental change period. The only self-reporting measure to assess the explicit phenotype of environmental sensitivity in children aged 8–19 years is the 12-item Highly Sensitive Child (HSC) Scale (11, 12, 24). It has been shown that this scale is a valid and reliable tool to directly describe and capture the hypothesized phenotype trait of environmental sensitivity in young people across different countries (25). The HSC scale comprises a bifactor model including a general factor and three specific factors: (1) Ease of Excitation (EOE), which represents how easily disturbed an individual is by internal and external influences; (2) Low Sensory Threshold (LST), which considers unpleasant sensory arousal to outside stimuli and low tolerance for these inputs; and (3) Aesthetic Sensitivity (AES), which captures aesthetic awareness. The HSC scale has been shown to capture children’s susceptibility to family environments, moderating the effects of high or low quality of parenting care on children’s depressive symptoms (26). To date, none of the previous studies have examined the psychometric properties of the HSC scale in Chinese adolescents, including depressed adolescents. Therefore, an important step for research in this area is to assess environmental sensitivity in Chinese adolescent that can identify an adolescent’s sensitivity to the living environment and its influence on depressive symptoms.

Adolescent depressive symptoms are often ignored by parents and be seen as an excuse for not performing academic well or not being obedient (27). Previous research has revealed that maternal support and behavioral control, comparing with paternal warmth (28–31), had stronger associations with adolescent depression. However, more recent studies have indicated that the direct effect of maternal warmth in reducing risk for adolescent depression was small but non-trivial (32). Some researchers have suggested that the interaction of parenting behaviors with other adolescent factors may be particularly important for the development of depression symptoms in adolescents (17). Many existing works have shown that the different levels of environmental sensitivity moderated caregiving environments affecting depressive symptoms in adolescents and adults (33, 34). The traditional diathesis-stress model holds that environmental sensitivity is a risk factor for adolescent mental health; on the contrary, the differential susceptibility theory seeks to present an organizational structure for understanding the supportive effect of ES in terms of buffering depression. In the current study, it was first hypothesized that highly sensitive Chinese adolescents may not just have an increased risk of depression with low-quality maternal behaviors, but also benefit from high-quality maternal behaviors and have opportunities for recovering from depressive symptoms. Second, maternal behaviors might not have an important role to play in the development of depression among low ES groups. One epidemiological study showed that the rates of adolescent depression in western China was higher than other areas (4). Hence, Shaanxi province targeted in this research was appropriate for investigating the associations of environmental sensitivity with depressive symptoms among Chinese adolescents. To our knowledge, no previous studies have clarified the effect of the relationship of the two factors (maternal behaviors and environmental sensitivity) on adolescent depressive symptoms.

In this study, we aimed to: (1) verify whether the HSC scale is a good fit for Chinese adolescents; (2) test for the HSC scale’s measurement invariance within genders and different groups; and (3) based on the differential susceptibility theory, investigate how environmental sensitivity moderates the association between maternal behaviors and Chinese adolescent depressive symptoms.

## Materials and methods

### Participants and procedure

This study involved two samples. A healthy group sample included 2,166 adolescents who were recruited from four secondary schools in Xi’an, China between December 2018 and March 2019. They included 744 boys and 1,422 girls. The mean age of the adolescents was 13.74 years (SD = 1.46, range = 12–17 years). A translation of the HSC scale was approved by the first author of the HSC scale. Schools and parents were asked to provide informed consent. Adolescent assent was asked to be provided via the first page at the beginning of the online survey (wjx.cn), when participants ticked the button “I understand and agree to be in the study.” The participants were required to finish questionnaires online at their school in a psychology class conducted by trained research assistants without teachers present. Regarding the depression group, a total of 105 adolescents with depression were recruited from outpatient clinics and inpatient ward from the First Affiliated Hospital of Xi’an Jiaotong University (ages ranged from 14 to 17 years) between 2018 and 2019. A diagnosis of depression in adolescents was ascertained using the 5th edition of the Diagnostic and Statistical Manual of Mental Disorders (DSM-V) based on a diagnostic structured interview, and then parents and patient consent was obtained via doctors after being told the purpose of the study. Participants completed background information gathering and all assessments in a psychometric room at the hospital. Participants with other psychotic disorders, head injury, and neurological diseases were excluded from this study. Ethical approval was obtained from the Ethics Committee of the First Affiliated Hospital, Xi’an Jiaotong University (No. 81771471).

### Measures

#### Demographic questionnaire

Data on demographic information included sex, grade, age, body mass index (BMI), mother’s and father’s education, only child status, and family income.

#### Highly Sensitive Child Scale-Chinese version

The Highly Sensitive Child Scale (HSC-12) investigates environmental sensitivity in children (35). It comprises 12 items and three subscales: EOE, LST, and ASE. Each item was rated on a 7-point scale ranging from 1 (not at all) to 7 (extremely), with higher scores indicating more sensitivity to the environment. The construct validity, internal reliability, and factor structure of the scale was good in adolescent samples from the United Kingdom (25).

The HSC scale with 12 items was translated into the Chinese language by a bilingual translator. Then another translator back translated it into English to guarantee accuracy. Two psychology and two psychiatry experts then explored each item that may have cultural and linguistic differences, and attempted to reduce these inequivalences. The HSC scale was then piloted for acceptability in 50 adolescents and, according to the participant feedback, the scale was expressed clearly and had cultural equivalence.

#### Parental bonding instrument

The 7-item Parental Bonding Instrument (PBI-7) is a self-administrated scale measuring maternal behavior (36), which comprises of two subscales: care and overprotection. Maternal care included 3-items, higher scores indicating that the mother makes her children feel more warmth and closeness. Maternal overprotection included 4-items, with high scores indicating that the mother is overinvolved in the adolescent’s events and prevents their independence. It is measured by a 4-point scale ranging from 1 (strongly disagree) to 4 (strongly agree). The PBI-7 has demonstrated good internal consistency.

#### Patient health questionnaire

The 9-item Patient Health Questionnaire (PHQ-9) was used to assess the existence and severity of symptoms of depression in the previous 2 weeks, rated by a 4-point scale ranging from 0 (*not at all*) to 3 (*nearly every day*). This scale is reported to have good psychometric properties and has been widely used to Leung et al. (27).

#### Sleep

Sleep duration: Participants were asked how many hours they slept in the preceding night. The hours of sleep duration ranged from 4 to 9 h (37).

#### Academic achievement

Academic achievement was determined by adolescent school grades for three major subjects (Chinese, Mathematics, and English). The total score of the three subjects for each adolescent was averaged for composite score analysis (38).

### Statistical analysis

**FIGURE 1:**
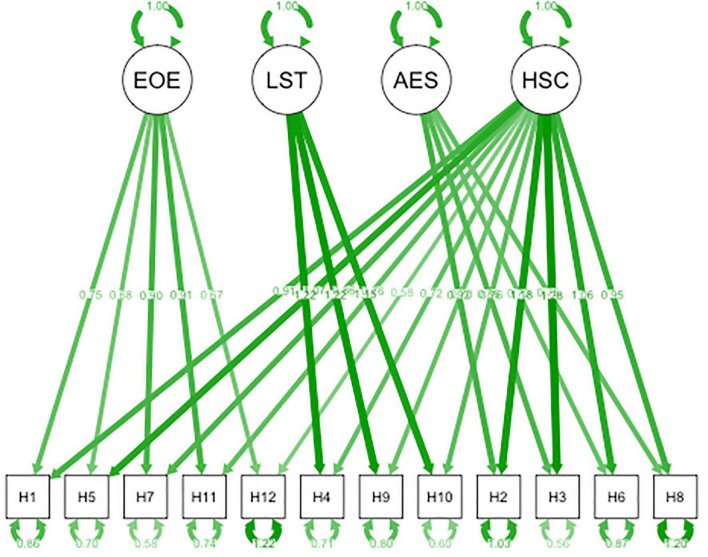
Bifactor model of the 12-item version of the Highly Sensitive Child (HSC) Scale.

First, to explore the underlying factor structure of the HSC scale in Chinese adolescents, exploratory factor analysis (EFA) by direct oblimin rotation was applied to 50% of the healthy sample, verifying whether each item would load on one of the three specific factors that were found by Pluess et al. The criteria for acceptance of the factor structures were based on the Bartlett test, *p* values of 0.005 or below, and a Keyser–Meyer–Olkin (KMO) value of 0.06 or above, with each factor including at least three items that loaded with a value of 0.40 or higher. Second, confirmatory factor analysis (CFA) was performed to investigate if we could replicate the bifactor model of the HSC scale in the other 50% of the healthy sample (see Figure 1). The CFA was conducted in R (lavaan and semPlot packages) using the maximum likelihood robust estimation method. Model fit was considered satisfactory if the comparative fit index and the Tucker Lewis index were stronger than 0.95 and 0.97, respectively, the root mean square error of approximation ranged between 0.05 and 0.08, and the standardized root mean square residual was 0.08 or below (35). Third, internal consistency of the HSC scale was evaluated by Cronbach’s α. Item-total and inter-item correlations were computed by Pearson’s correlation coefficients. Fourth, we tested measurement invariance across the following characteristics: (a) girls versus boys, and (b) depressive versus healthy adolescents. When measurement invariance is established, the fit indicators of the HSC scale model: ΔCFI ≤ 0.01 and ΔRMSEA ≤ 0.015, should not significantly worsen according to every tighter constraint that has been made (39).

*T*-test and chi-square tests were performed to examine the significance of variables between healthy and depression groups. Subsequently, the Pearson correlations were calculated between environmental sensitivity, maternal behavior, and adolescent depressive symptoms.

With regard to the moderation model, the interaction between maternal behaviors and environmental sensitivity on depressive symptoms were tested by simple slope analysis using R (jtools, interactions and lm.beta). Then using the RoS method to test the diathesis-stress model, and differential susceptibility model (40).

## Results

### Construct validity and measurement invariance

Using the EFA, the KMO value of the HSC scale was 0.891 and Bartlett’s test of Sphericity was 4,321.088, which was statistically significant (*p* < 0.01). The EFA found three principal components explained 73.55% of the cumulative variance. Item loadings of the three subscales were stronger than 0.40 (see Table 1).

**TABLE 1:** Exploratory factor analysis of the Highly Sensitive Child (HSC) Scale.

	Factor		
	
Items	1 (EOE)	2 (AES)	3 (LST)
(1) I find it unpleasant to have a lot going on at once	**0.77**	0.16	–0.03
(2) Some music can make me really happy	0.06	**0.84**	0.04
(3) I have nice tastes	–0.08	**0.87**	–0.02
(4) Loud noises make me feel uncomfortable	0.28	0.25	**0.87**
(5) I am annoyed when people try to get me to do too many things at once	**0.73**	0.22	0.01
(6) I notice it when small things have changed in my environment	0.04	**0.80**	0.21
(7) I get nervous when I have to do a lot in little time	**0.87**	0.12	0.16
(8) I love nice smells	0.19	**0.75**	0.13
(9) I don’t like watching TV programs that have a lot of violence in them	–0.13	–0.01	**0.85**
(10) I don’t like loud noises	0.14	0.24	**0.86**
(11) I don’t like it when things change in my life	**0.84**	–0.15	0.32
(12) When someone observes me, I get nervous. This makes me perform worse than normal	**0.65**	–0.13	0.00

EOE, ease of excitation; AES, aesthetic sensitivity; LST, low sensory threshold. Bold values mean factors included at least three items that loaded with a value of 0.40 or higher.

Confirmatory factor analysis showed that three models were tested, the three factors found in the EFA (Model 1), the bifactors found by the authors of the scale (Model 2), a model with the three first-order factors and one second-order factor (Model 3), Table 2 shows the three models. The fit indices for the bifactor model of the HSC scale were CFI = 0.989, GFI = 0.977, AGFI = 0.958, TLI = 0.983, RMSEA = 0.042, and RMR = 0.050. Three principal components were composed of 12 selected items, including EOE (items 1, 5, 7, 11, and 12), AES (items 2, 3, 6, and 8) and LST (items 4, 9, and 10). Figure 1 shows the completely standardized factor loadings for Model 2.

**TABLE 2:** Fit indices of the Highly Sensitive Child (HSC) Scale.

Model	CFI	GFI	AGFI	TLI	RMSEA	RMR
Model 1	0.901	0.884	0.832	0.879	0.114	0.658
Mode 2	0.989	0.977	0.958	0.983	0.042	0.05
Model 3	0.984	0.969	0.953	0.980	0.047	0.072

Model 1: Three factors found from EFA, Model 2: Bifactors proposed by the scale’s authors, Model 3: One second-order factor and three first-order factors found by EFA.

The bifactor model with correlated item residuals for the 12 HSC items fit the data well and the factor structure (configural), factor loadings (metric), item intercept (scalar), and error variances (strict) were invariant across different samples (see Table 3).

**TABLE 3:** Measurement invariance of the Highly Sensitive Child (HSC) Scale across gender and clinical group.

	χ2 (df)	RMSEA	CFI	TLI	*p*	Δ CFI
***Boys versus girls***						
Configural	123.4 (72)	0.048	0.988	0.978	–	–
Metric	139.31 (92)	0.041	0.989	0.984	0.72	–0.001
Scalar	148.41 (100)	0.040	0.989	0.985	0.33	0.000
strict	168.51 (112)	0.041	0.987	0.985	0.07	0.002
***Health versus depression***						
Configural	123.40 (72)	0.048	0.988	0.978	–	–
Metric	152.48 (92)	0.043	0.988	0.983	0.09	0.000
Scalar	158.83 (100)	0.041	0.988	0.985	0.61	0.000
strict	179.21 (112)	0.04	0.987	0.985	0.06	0.001

### Reliability and item characteristics

The HSC scale showed adequate internal consistency with α = 0.892 and each factor ranged from 0.879 to 0.887. The item to total correlations varied from *r* = 0.464 to *r* = 0.735 (see Table 4). The inter-item correlations ranged between *r* = 0.167 and *r* = 0.740.

**TABLE 4:** Each item mean score and reliability of the Highly Sensitive Child (HSC) Scale.

Items	X ± S	Cronbach’s α if item deleted	Corrected item-to-total correlation
H1	3.29 ± 1.506	0.883	0.627
H2	3.22 ± 1.811	0.882	0.638
H3	3.38 ± 1.715	0.887	0.735
H4	3.70 ± 1.645	0.889	0.511
H5	3.66 ± 1.521	0.879	0.710
H6	3.51 ± 1.575	0.881	0.659
H7	3.59 ± 1.463	0.882	0.653
H8	3.51 ± 1.621	0.885	0.593
H9	3.56 ± 1.665	0.890	0.504
H10	3.67 ± 1.580	0.887	0.554
H11	3.49 ± 1.468	0.885	0.598
H12	3.69 ± 1.417	0.891	0.464

### Sample characteristics and correlation analysis

Participant characteristics are shown in Table 5. Depressive adolescents had significantly higher scores on the HSC-total (*F* = 15.11, *p* < 0.01) and HSC-EOE (*F* = 37.84, *p* < 0.01), but lower scores on AES (*F* = 6.31, *p* < 0.01) when compared to other groups. According to multivariate analysis of variance, the three groups differed significantly regarding maternal behaviors (*F* = 36.30, *p* < 0.01; *F* = 41.56, *p* < 0.01), with post hoc tests showing that maternal care was significantly lower and overprotection was higher in the depressive group.

**TABLE 5:** Characterization and differences among subgroups.

	Control		Depression adolescent	*F/t*	*p*
			
	Early adolescent *N* = 1,132	Middle to late adolescent *N* = 609	*N* = 105		
Age [mean (SD)]	12.81 (0.70)	15.47 (0.78)	16.3 (1.64)		
Sex	54.68% boys	53.20 boys	30.48 boys		
Grade	7–9	10–12	7–12		
HSC	5.00 (0.66)	5.04 (0.73)	5.38 (0.65)	15.11	<0.01
HSC-EOE	4.58 (1.00)	4.57 (0.98)	5.46 (1.10)	37.84	<0.01
HSC-LST	5.25 (1.12)	5.25 (1.13)	5.47 (0.96)	1.89	0.15
HSC-AES	5.34 (0.84)	5.47 (0.95)	5.23 (0.84)	6.31	<0.01
Maternal care	2.14 (0.55)	2.24 (0.52)	1.73 (0.68)	36.30	<0.01
Maternal overprotection	1.05 (0.52)	0.96 (0.49)	1.46 (0.68)	41.56	<0.01
School night sleep duration	6.50 (1.14)	5.82 (1.14)		12.70	<0.01
PHQ-9	7.14 (4.96)	6.92 (5.17)		0.82	0.41

Table 6 revealed that ES was significantly positively associated with maternal care (*r* = 0.18, *p* < 0.01) and negatively correlated with maternal overprotection (*r* = −0.12, *p* < 0.01). Depressive symptoms (depressive emotion, sleep duration, and academic performance) were not significantly correlated with ES, indicating that ES did not mediate the effects of maternal behaviors on adolescent depressive symptoms. We therefore analyzed ES as a moderator.

**TABLE 6:** Correlations between study variables.

Measure	1	2	3	4	5	6	7	8
(1) HSC	–							
(2) Maternal care	0.18**	–						
(3) Maternal overprotection	−0.12**	−0.47**	–					
(4) PHQ-9	0.05	−0.33**	0.28**	–				
(5) Age	0.01	0.004	0.004	–0.03	–			
(6) Gender	–0.03	0.04	−0.08*	0.02	−0.19**	–		
(7) School night sleep duration	0.05	0.12**	−0.10*	−0.22**	0.06	–0.04	–	
(8) Academic performance	–0.06	–0.02	–0.08	−0.15**	−0.16**	0.04	−0.08*	–

*p < 0.05; **p < 0.01.

### Moderating effects of environmental sensitivity

As shown in Table 7, the *R*^2^ for the endogenous variables indicated that the model counted for 9.7% of the variance in depression, 1.5% in sleep, and 13% in academic performance. The moderating effect of environmental sensitivity on relationship between maternal behaviors and adolescent depressive symptoms were significant.

**TABLE 7:** Summery of regression model.

	Depression *R*^2^ adjusted:13.1		Sleep *R*^2^ adjusted:1.6		Academic *R*^2^ adjusted:18.3	
			
Variables	*b*	*p*	*b*	*p*	*b*	*p*
Environmental sensitivity	0.056	0.02	–0.05	0.233	0.169	0.00
PBIM care	–0.22	0.00	0.089	0.05	0.317	0.00
PBIM overprotection	0.147	0.00	–0.056	0.219	0.016	0.707
Environmental sensitivity × PBIM care	–0.15	0.001	–	–	0.188	0.000
Environmental sensitivity × PBIM overprotection	0.139	0.024	–	–	–0.098	0.013

The model is adjusted for covariates (sex, age, and presence of siblings).

**FIGURE 2:**
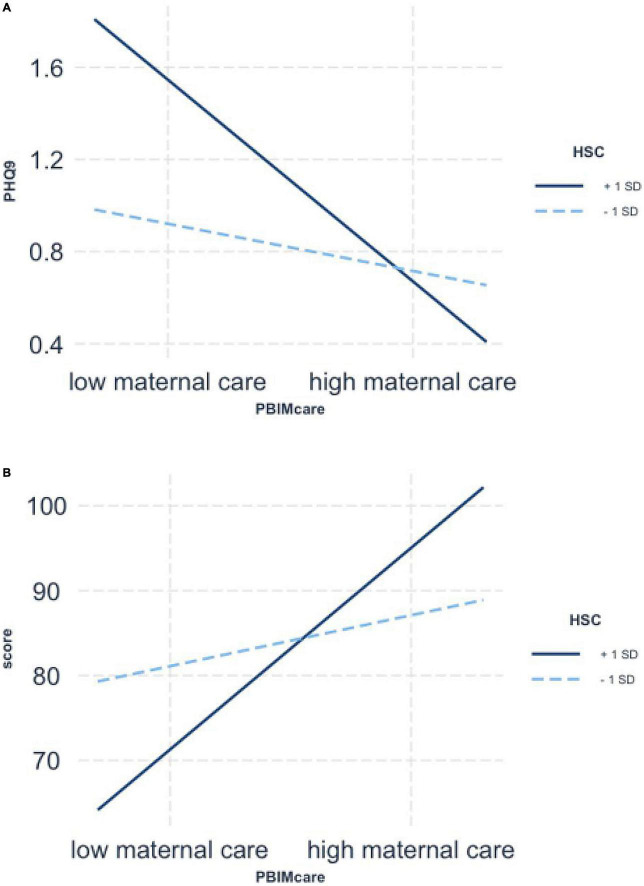
Simple slopes for moderated regression of Highly Sensitive Child (HSC) (1 SD above and below the mean) and maternal care interaction predicting adolescent’s depression **(A)** and academic scores **(B)**.

**FIGURE 3:**
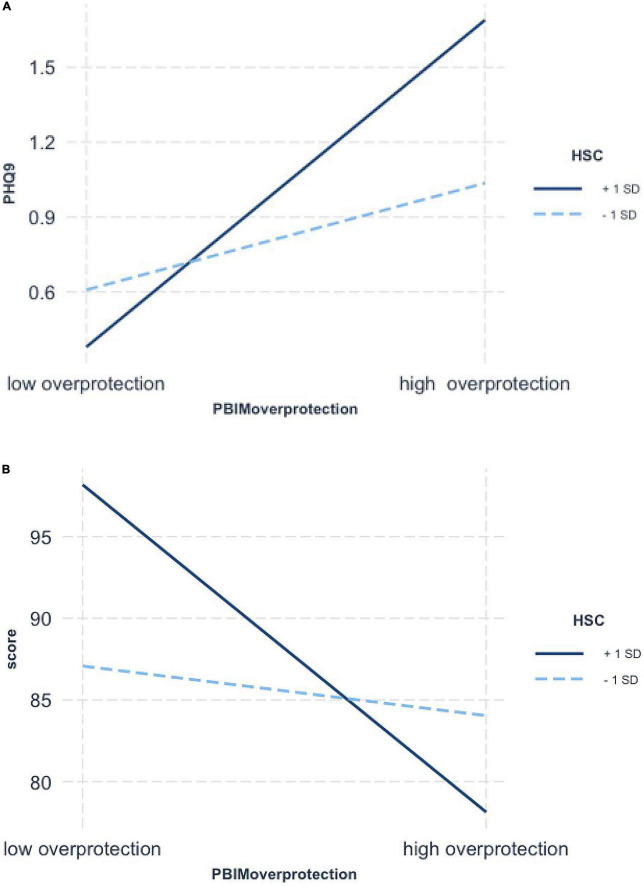
Simple slopes for moderated regression of Highly Sensitive Child (HSC) (1 SD above and below the mean) and maternal overprotection interaction predicting adolescent’s depression **(A)** and academic scores **(B)**.

Figure 2A shows that the negative association between maternal care and depression was greater in the highly sensitive group (+1 *SD*) (*b* = −0.52, *SE* = 0.06, *t* = −8.87, *p* < 0.001), than the low sensitive group (−1 *SD*) (*b* = −0.32, *SE* = 0.04, *t* = −7.50, *p* < 0.001). Moreover, with reference to the second interaction effect, Figure 2B shows that experiencing higher quality of maternal care was significantly associated with higher scores in academic achievement only when adolescent sensitivity was high (*b* = 14.24, *SE* = 1.47, *t* = 9.71, *p* < 0.001). By comparison, the relation was not significant (*b* = 3.58, *SE* = 1.46, *t* = 2.45, *p* > 0.05) when it came to low sensitivity scores. Figure 3A shows that maternal negative control and overprotection were significantly associated with adolescent depression if sensitivity was high (*b* = 0.44, *t* = 7.48, *SE* = 0.06, *p* < 0.001), but not if sensitivity was low (*b* = 0.14, *t* = 2.21, *SE* = 0.06, *p* = 0.03). Furthermore, simple slope analysis revealed that high ES (+1 *SD*) adolescent’s academic scores were significantly affected by maternal overprotection (*b* = −6.68, *SE* = 1.51, *t* = −4.43, *p* < 0.001) whereas low ES (−1 *SD*) adolescent’s scores were not significantly affected by maternal overprotection (*b* = −1.01, *SE* = 1.67, *t* = −0.61, *p* = 0.55; see Figure 3B). However, no interaction was found between maternal behaviors and environmental sensitivity on adolescents’ sleep duration.

### Identification of environmental sensitivity patterns

**FIGURE 4:**
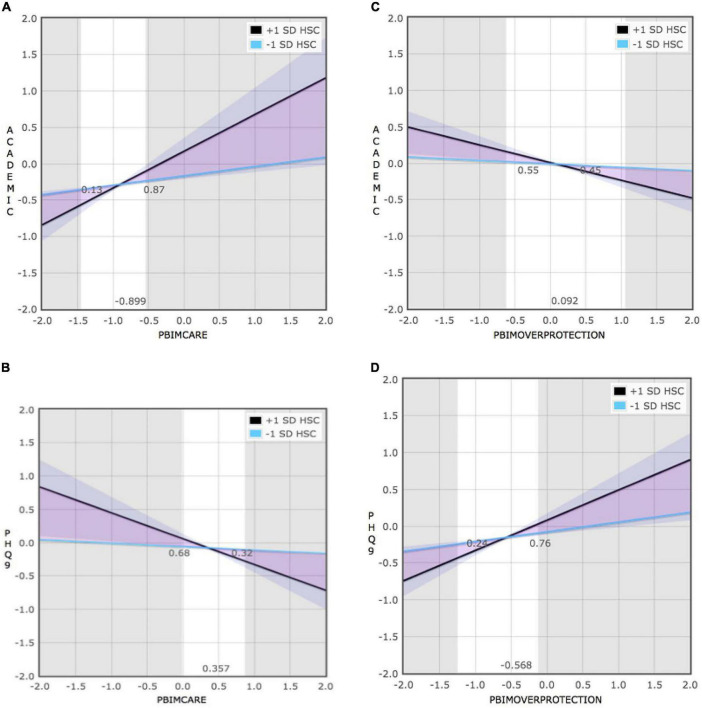
Linear regression of adolescent depression and academic to maternal behaviors **(A–D)**.

Simple slope analysis showed that the interaction between maternal behaviors and environmental sensitivity was significant in predicting adolescent depression and academic performance, and then the RoS method was employed to further assess which theoretical model the interaction between environmental sensitivity and maternal behaviors is consistent with. The results are reported in Table 8 and Figure 4. According to the indicators, the interaction between maternal behaviors and environmental sensitivity on adolescent depression and academic conform to the Differential Susceptibility model.

**TABLE 8:** Summary of RoS of interaction between maternal behaviors and environmental sensitivity on adolescent academic and depression.

Result	RoS X		PoI	PA	Intersection
				
	Lower boundary	Upper boundary			
PBIM care × HSC on academic	−1.454	–0.540	0.87	0.816	–0.899
PBIM care × HSC on depression	−0.012	0.871	0.31	0.345	0.357
PBIM overprotection × HSC on academic	−0.619	1.059	0.45	0.463	0.092
PBIM overprotection × HSC on depression	−1.246	–0.124	0.76	0.715	–0.568

## Discussion

It is of great importance to focus on understanding environmental sensitivity and whether it may contribute to increased or decreased adolescent depressive symptoms. The current study is the first to test the psychometric properties of the Highly Sensitive Child Scale and its invariance across gender and clinical groups in Chinese adolescents. The findings support a bifactor structure of the Chinese version of the HSC scale in this population. Furthermore, the study explored the moderating role of environmental sensitivity as a potential mechanism explaining the association between maternal behaviors and adolescent depressive symptoms in Chinese adolescents. Adolescents with high environmental sensitivity may have potential for outperformance when they experience supportive parenting, however, those scoring high on environmental sensitivity in association with low-quality parenting are often correlated with depressive symptoms. Maternal behaviors significantly predicted adolescents’ depressive symptoms when their environmental sensitivity was high. In contrast, this effect become non-significant when adolescents’ environmental sensitivity was low.

In the current study, the Chinese version of the HSC scale showed good internal consistency (α of 0.892) in which all subscales exceed 0.70. Results from EFA and CFA indicated that HSC-CV was a bifactor structure to assess environmental sensitivity in Chinese adolescents with three factors accounting for 73.55% of the total variance and the load of the 12 items exceeding 0.40, which was similar to that reported in previous studies (41). Measurement invariance implied that the HSC-CV was measuring the same construct across boys and girls as well as healthy and depressive samples, which is the first to do so in Chinese adolescents.

The study findings suggest that depressive adolescents, compared with a healthy group, focused greater on surrounding environments, being more vulnerable to the adverse effects of family influences but gaining less benefit from positive environmental exposures. A possible explanation for this is that depressive adolescents always take negative stimuli from their environment, which could in turn drive them to adjust their negative environmental susceptibility, ignoring positive resources in their daily life. This may explain why depressed adolescents experience only small improvements following psychological therapy (42), with less sensitivity to experience good feelings in association with an intervention. The risk of depressive relapse among adolescents in remission is high (43). This suggests that adolescents should sometimes avoid over stimulating environments, have enough time to focus on themselves and process their things, and have a supportive individual with normal susceptibility to help them. Regarding the variables of maternal behaviors and environmental sensitivity, senior high school students scored better than junior high school students, suggesting that when children are older they have more ability to resolve social, emotional, and academic challenges despite being at the same level of environmental sensitivity. Thus, younger students with high environmental sensitivity may be a risk population for future mental problems, and we should thus focus on depressive symptoms in junior high students.

The current study indicated that maternal behaviors predicted adolescents’ depressive symptoms, however, these effects were weak, consistent with a large body of previous studies (17) indicating that although mothers would change themselves in a good way, adolescents may not feel better as a result. The current study went on to examine whether environmental sensitivity moderated the relationship between maternal behaviors and adolescents’ depressive symptoms. Although prior study has indicated that environmental sensitivity significantly impacts depressive symptoms, to our knowledge this study is the first to identify that high environmental sensitivity can significantly strengthen not only the adverse effect of maternal behaviors on Chinese adolescents’ depressive symptoms but also the positive effect of maternal behaviors on well-being. Specifically, for adolescents with high environmental sensitivity, high-quality maternal behaviors protected against depressive emotions and poor academic performance, and low-quality maternal behaviors were associated with worse depressive symptoms. In contrast, for adolescents with fewer susceptibilities, maternal care and overprotection were unrelated to depressive symptoms. These finding are in line with previous studies on externalizing behavior, physical health, and social well-being in children that have documented stronger protective effects of high-quality parenting among children with high environmental sensitivity, support the differential susceptibility model (44, 45). Adolescent depressive symptoms might be particularly vulnerable to the influence of maternal behaviors and environmental sensitivity simultaneously. It is noteworthy that moderating effects were most pronounced for high sensitivity adolescents.

The findings revealed that environmental sensitivity in adolescents had no moderating effect on the association between maternal behaviors and sleep duration, and this is not in keeping with previous studies found that highly susceptible children showed more sleeping problems compared with children with average or low susceptibility (46). The reason for this discrepancy might be that when Chinese adolescents are faced with more schoolwork, they gradually adopt a pattern of short sleep duration on school nights; our data showed that junior high school students averaged 6.5 h of sleep per school night, while senior high school students averaged just 5.82 h, approximately 2–4 h less than National Sleep Foundation’s recommendation of 8–10 h (47). Hence, groups with higher or lower susceptibility showed no difference in sleep problems.

The current study has multiple strengths. To our knowledge, our research is the first to validate the HSC scale in the Chinese adolescents and then apply this scale to a depressive sample. However, the findings should be considered in light of some limitations. First, all of the related data are from one self-reported psychological indicator of environmental sensitivity. Further studies should have similar analyses to identify other, more biological markers and expand the research to cover children aged 7–12 years. Second, the cross-sectional nature of the study limited our ability to determine causal inferences and a prospective study measuring ES at different stages of adolescent development may help us better understand changes in ES. Lastly, lacking another personality scale calculated the convergent validity, however, its inclusion would increase the testing time in order to reduce the reliability of the study. Future research should focus on the relationship between other personalities and environmental sensitivity.

## Conclusion

The HSC scale has good reliability and validity to capture Chinese adolescent abilities to register and process environmental factors. Furthermore, Chinese adolescents perceiving maternal behaviors are qualitatively different at varying levels of environmental sensitivity, and the positive and negative aspects of maternal function differently in relation to depression at high levels of sensitivity. The current study shows that environmental sensitivity may be a potential personality effect factor for the development of depressive symptoms in Chinese adolescents. Environmental sensitivity as a predictor of the effectiveness of interventions for depression may facilitate the design of personalized treatments for sensitive and less sensitive adolescents.

## Data availability statement

The raw data supporting the conclusions of this article will be made available by the authors, without undue reservation.

## Ethics statement

The studies involving human participants were reviewed and approved by the Ethics Committee of The First Affiliated Hospital, Xi’an Jiaotong University. Written informed consent to participate in this study was provided by the participants’ legal guardian/next of kin.

## Author contributions

QD performed the literature studies, designed the research, and wrote the manuscript. LZ conducted statistical analysis and reviewed the research. WW recruited the subjects and reviewed the research. XW recruited the subjects. MP provided the research tool. XM designed, reviewed, and supervised the research. All authors have contributed to and have approved the final manuscript.
